# Supplementary material for: Malignant neoplasms in people with hypothyroidism in Spain: A population-based analysis
Source: PLoS One. 2022 Oct 5;17(10):e0275568. doi: 10.1371/journal.pone.0275568 (PMC9534429; doi:10.1371/journal.pone.0275568)
Supplement: S1 Table — (DOCX) [file pone.0275568.s001.docx]

**Table S1. Prevalence of the different malignant neoplasms studied in the Spanish population in 2019**

|  |  | **Gender** | | **Age (years)** | | | |
| --- | --- | --- | --- | --- | --- | --- | --- |
|  | **All** | **Male** | **Female** | **0-14** | **15-34** | **35-64** | **65 and over** |
| **All malignancies** | 1,819,173 (4.74) | 865,161 (4.75) | 954,013 (4.74) | 14,259 (0.25) | 51,558 (0.68) | 649,489 (3.79) | 1,103,868 (13.70) |
| **Breast** | 301,853 (1.50) |  | 301,853 (1.50) |  | 1,800 (0.05) | 142,881 (1.61) | 157,026 (3.41) |
| **Colorectal** | 255,339 (0.67) | 140,177 (0.77) | 115,162 (0.57) | 427 (0.01) | 1,944 (0.03) | 76,151 (0.44) | 176,817 (2.19) |
| **Prostate** | 224,925 (1.23) | 224,925 (1.23) |  |  |  | 29,843 (0.36) | 194,993 (5.66) |
| **Hematologic** | 149,375 (0.39) | 75,691 (0.42) | 73,684 (0.37) | 5,843 (0.10) | 18,608 (0.25) | 61,664 (0.36) | 63,260 (0.79) |
| **Respiratory tract** | 104,840 (0.27) | 73,933 (0.41) | 30,907 (0.15) | 758 (0.01) | 1,952 (0.03) | 40,311 (0.24) | 61,819 (0.77) |
| **Bladder** | 104,089 (0.27) | 84,256 (0.46) | 19,833 (0.10) |  | 541 (0.01) | 24,579 (0.14) | 78,858 (0.98) |
| **Thyroid** | 43,466 (0.11) | 9,094 (0.05) | 34,372 (0.17) |  | 2,657 (0.04) | 26,820 (0.16) | 13,878 (0.17) |
| **Digestive (other)** | 43,421 (0.11) | 27,313 (0.15) | 16,108 (0.08) |  | 665 (0.01) | 16,366 (0.10) | 26,249 (0.33) |
| **Renal** | 39,980 (0.10) | 24,992 (0.14) | 14,989 (0.07) | 493 (0.01) | 642 (0.01) | 13,705 (0.08) | 25,140 (0.31) |
| **Cervix** | 38,415 (0.19) |  | 38,415 (0.19) |  | 3,490 (0.09) | 26,889 (0.30) | 8,027 (0.17) |
| **Gastric** | 24,759 (0.06) | 13,991 (0.08) | 10,769 (0.05) |  |  | 6,794 (0.04) | 17,688 (0.22) |
| **Pancreas** | 14,497 (0.04) | 7,307 (0.04) | 7,190 (0.04) |  |  | 4,717 (0.03) | 9,643 (0.12) |

Data are the absolute values of patients with malignancies and the percentage with respect to the total number of people in their group of gender or age.

The percentages of breast and cervical cancer have been calculated on the total population of Spanish women (20,134,521). The percentage of prostate cancer is calculated on the total male population (18,230,737).
