# Supplementary material for: Malignant neoplasms in people with hypothyroidism in Spain: A population-based analysis
Source: PLoS One. 2022 Oct 5;17(10):e0275568. doi: 10.1371/journal.pone.0275568 (PMC9534429; doi:10.1371/journal.pone.0275568)
Supplement: S2 Table — (DOCX) [file pone.0275568.s002.docx]

**Table S2. Relative risk of the different site-specific malignancies studied in patients with hypothyroidism classified according to socioeconomic variables**

|  | Breast | Colorectal | Prostate | Hematological | Respìratory | Bladder | Thyroid | Digestive | Renal | Cervix | Gastic | Pancreas |
| --- | --- | --- | --- | --- | --- | --- | --- | --- | --- | --- | --- | --- |
| All | 1.49  1.48-1.51 | 1.42  1.40-1.44 | 1.76  1.72-1.80 | 1.74  1.71-1.77 | 1.83  1.79-1.87 | NS | 5.07  4.96-5.18 | 1.58  1.53-1.63 | 1.52  1.47-1.57 | 1.29  1.25-1.33 | 1.54  1.48-1.61 | 1.59  1.50-1.68 |
| Income level (€/year) | |  |  |  |  |  |  |  |  |  |  |  |
| ≥18,000 | 1.45  1.42-1.48 | 1.41  1.38-1.45 | 1.84  1.78-1.90 | 1.76  1.70-1.82 | 1.98  1.91-2.06 | NS | 4.42  4.24-4.60 | 1.47  1.37-1.57 | 1.69  1.59-1.79 | 1.49  1.40-1.59 | 1.47  1.34-1.60 | 1.37  1.23-1.53 |
| <18,000 | 1.50  1.48-1.52 | 1.41  1.38-1.43 | 1.67  1.62-1.72 | 1.75  1.72-1.79 | 1.78  1.74-1.83 | NS | 5.46  5.31-5.61 | 1.57  1.51-1.63 | 1.44  1.38-1.51 | 1.21  1.16-1.25 | 1.55  1.18-1.63 | 1.60  1.50-1.72 |
| Very low | 1.55  1.49-1.62 | 1.61  1.51-1.73 | NA | 1.57  1.45-1.69 | 1.58  1.45-1.72 | NA | 4.37  3.98-4.79 | 1.95  1.73-2.21 | NA | 1.26  1.13-1.41 | NA | NA |
| Municipality size (inhabitants) | |  |  |  |  |  |  |  |  |  |  |  |
| <10,000 | 1.53  1.48-1.57 | 1.39  1.34-1.44 | 1.66  1.57-1.75 | 1.69  1.62-1.77 | 1.83  1.74-1.93 | NS | 6.61  6.23-7.01 | 1.86  1.73-2.01 | 1.48  1.34-1.63 | 1.28  1.17-1.41 | 1.41  1.26-1.58 | NA |
| 10,000-50,000 | 1.53  1.51-1.56 | 1.35  1.31-1.38 | 2.05  1.97-2.13 | 1.72  1.67-1.78 | 1.94  1.87-2.02 | NS | 5.89  5.66-6.14 | 1.48  1.39-1.57 | 1.62  1.52-1.73 | 1.50  1.41-1.59 | 1.19  1.09-1.31 | 1.46  1.31-1.64 |
| 50,001-100,000 | 1.42  1.38-1.47 | 1.50  1.45-1.56 | 1.66  1.56-1.77 | 1.74  1.65-1.82 | 1.87  1.77-1.98 | 1.16  1.08-1.24 | 6.15  5.80-6.52 | 1.55  1.41-1.71 | 1.54  1.39-1.70 | 1.26  1.16-1.36 | 1.87  1.65-2.12 | NA |
| 100,001-500,000 | 1.44  1.41-1.47 | 1.41  1.38-1.45 | 1.53  1.47-1.60 | 1.59  1.54-1.65 | 1.67  1.60-1.73 | 0.92  0.89-0.97 | 4.58  4.39-4.77 | 1.58  1.49-1.69 | 1.32  1.23-1.41 | 1.18  1.11-1.26 | 1.47  1.35-1.60 | 1.66  1.49-1.84 |
| >500,000 | 1.48  1.44-1.51 | 1.43  1.39-1.47 | 1.77  1.69-1.85 | 2.02  1.94-2.09 | 1.80  1.73-1.87 | NS | 3.38  3.21-3.56 | 1.50  1.39-1.62 | 1.58  1.47-1.69 | 1.18  1.10-1.25 | 2.03  1.87-2.21 | 2.01  1.82-2.23 |
| Country of birth |  |  |  |  |  |  |  |  |  |  |  |  |
| Spain | 1.51  1.49-1.53 | 1.47  1.44-1.49 | 1.81  1.76-1.85 | 1.75  1.71-1.78 | 1.82  1.78-1.86 | NS | 4.93  4.81-5.06 | 1.55  1.50-1.61 | 1.50  1.44-1.56 | 1.37  1.32-1.42 | 1.65  1.57-1.73 | 1.59  1.49-1.69 |
| Other | 1.39  1.36-1.42 | 1.23  1.20-1.27 | 1.55  1.47-1.63 | 1.66  1.60-1.72 | 1.76  1.69-1.83 | 0.93  0.87-0.99 | 4.73  4.53-4.93 | 1.68  1.58-1.80 | 1.47  1.35-1.60 | NS | NS | NA |
| Employment situation | |  |  |  |  |  |  |  |  |  |  |  |
| Active | 1.07  1.05-1.10 | 1.22  1.18-1.27 | 1.18  1.08-1.30 | 1.60  1.55-1.65 | 1.68  1.59-1.78 | 0.89  0.83-0.97 | 5.02  4.85-5.19 | 1.20  1.09-1.32 | 0.81  0.73-0.90 | NS | 1.61  1.43-1.82 | NA |
| Non-active | 2.68  2.62-2.75 | 3.83  3.70-3.97 | NA | 2.48  2.38-2.58 | 4.14  3.90-4.40 | 3.03  2.78-3.31 | 7.76  7.33-8.21 | 3.39  3.08-3.73 | 4.31  3.99-4.67 | 2.40  2.22-2.60 | 4.65  4.19-5.16 | NA |
| Pensioners | 1.50  1.42-1.59 | 0.84  0.83-0.86 | NS | 1.27  1.24-1.30 | 1.19  1.16-1.21 | 0.62  0.60-0.64 | 3.58  3.44-3.71 | NS | NS | 1.08  1.03-1.14 | 0.88  0.83-0.93 | NS |

Data are OR and 95% CI. Abbreviations: NA, not available; NS, not significant.
